# The nexus between maternal antenatal care attendance, newborn postnatal care and neonatal mortality in India: a matched case-control study

**DOI:** 10.1186/s12884-024-06881-6

**Published:** 2024-10-22

**Authors:** Wahengbam Bigyananda Meitei, Abhishek Singh

**Affiliations:** Department of Public Health and Mortality Studies, International Institute for Population Sciences, Mumbai, 400088 India

**Keywords:** Antenatal care, Postnatal care, Neonatal mortality, Matched case-control, India

## Abstract

**Introduction:**

Our study examines the relationship between newborn postnatal care and neonatal mortality stratified by maternal antenatal care attendance under a matched case-control framework.

**Methods:**

Data from the fifth round of the National Family Health Survey was used. A total of 172,079 recent births to eligible women (15–49 years) in five years preceding the survey were included in the study. We used the conditional logistic regression model, a commonly used regression model to fit matched case-control data to examine the effects of newborn postnatal care on neonatal mortality. The mother’s age at birth of the newborn, previous birth intervals, birth order of the newborn, and birthsize of the newborn were included as the matching variables.

**Results:**

Newborns receiving postnatal care within two days or more than two days of birth are less likely to die during the neonatal period. Preferences for newborn postnatal care were also observed to increase with more maternal antenatal care visits. Our study also found a lower risk of neonatal mortality among those newborns whose umbilical cord was examined within two days of birth, regardless of the number of maternal antenatal care visits. Similarly, the risk of newborn deaths was lower among babies whose body temperature was measured within two days of birth. The tendency to breastfeed their newborns within an hour after delivery was considerably higher among those births that occurred to mothers who had a higher number of maternal antenatal care visits. The risk of newborn deaths was also observed to be lower among those born in public or private healthcare facilities.

**Conclusion:**

Considering the cohesive nature of the relationship between neonatal mortality and maternal and child healthcare utilisation, strategic planning and management of the existing policies and programmes related to accessibility, availability, and affordability of maternal and child healthcare services is needed to achieve goal 3.2 of the Sustainable Development Goals. Promoting cost-effective measures such as continuous monitoring of the baby’s body temperature and umbilical cord care could also effectively help reduce neonatal mortality.

**Supplementary Information:**

The online version contains supplementary material available at 10.1186/s12884-024-06881-6.

## Introduction

The global under-five mortality has declined remarkably over the past few decades [1, 2]. However, at least four million newborns die within one month of life, of which 75% die within a week [3, 4]. Low-and-middle-income countries (LMICs) hold the highest proportion of neonatal and under-five deaths due to poor access to quality healthcare [4]. A substantial reduction in neonatal mortality could relieve the burden of under-five mortality in LMICs [2].

About 40% of neonatal deaths in India occur on the first day of life and 57% within the first three days [5]. One-fourth of all global neonatal deaths occurred in India [2, 4, 6]. India’s neonatal mortality trends depict a sluggish pace of decline from 49 deaths per 1,000 live births in 1992-93 to 25 deaths per 1,000 live births in 2019-21 [7]. Moreover, the contribution of neonatal mortality to under-five mortality has also increased from 45% in 1992-93 to 60% in 2015-16 and remains the same until 2019-21 [7]. With this high rate of neonatal mortality, addressing newborn health and achieving goal 3.2 of the Sustainable Development Goals (SDGs), which aims at achieving a neonatal mortality rate of 12 per 1,000 live births [8], remains a menacing challenge for the country [9].

Policies and programs promoting women to attend four or more antenatal care (ANC) visits and institutional deliveries have been operationalised in India over the past two decades [10, 11]. The Janani Sishu Suraksha Karyakram (JSSK) (an initiative promoting institutional delivery offering free and cashless services for both normal and caesarean deliveries, as well as free treatment of sick newborns for thirty days after birth in public hospitals both in rural and urban areas), introduced as a part of it, has dramatically increased institutional deliveries (89% in 2019-21) [7, 10]. However, in India, only 59% of women aged 15–49 had four or more ANC visits, and 61% of the mothers had postnatal care (PNC) within two days after delivery [7], suggesting the unequal distribution of healthcare access during the reproductive period. Although promoting PNC, ANC, and institutional deliveries are equally important, attendance of PNC appears to have received less attention than ANC and delivery care. While regular ANC visits can help in the early detection of obstetric conditions that may lead to ill effects on the newborn’s health [12], studies have also acknowledged the potential of PNC in reducing maternal and newborn deaths [11, 13].

The newborns first month is crucial as most deaths occur during delivery or in the immediate postpartum period [11, 13]. Most newborns in LMICs die without skilled care, and these deaths can be averted by providing adequate care to both mother and baby during pregnancy, delivery and the postpartum period [14].

The three relationships, namely, the number of maternal ANC visits and PNC utilisation [15–20], maternal ANC visits and neonatal mortality [12, 21–24], and PNC utilisation and neonatal mortality [11, 13, 20] in LMICs, have been studied separately quite extensively. However, there is limited or no evidence on how the relationship between newborn PNC utilisation behaviour and neonatal deaths varies by the number of maternal ANC visits. Understanding the cohesive relationship between maternal and newborn healthcare utilisation in different stages, from pregnancy to the postpartum period, affecting the newborns’ health, resulting in death, is necessary to help reduce neonatal mortality in India. Indeed, LMICs needs to improve the quality and coverage of ANC and PNC interventions simultaneously to achieve the SDG goals [15]. Thus, our study aims to study the impact of newborn PNC utilisation on neonatal mortality stratified by maternal ANC visits.

## Methods and materials

### Data

We used the fifth National Family Health Survey (NFHS-5) data. NFHS-5 is a nationally representative survey conducted in 2019-21, collecting information on maternal and child health indicators from all 36 states and union territories of India. The survey used a two-stage stratified sampling method where 30,456 primary sampling units were included and interviewed 724,115 women aged 15–49 years from 636,699 surveyed households with a response rate of 97%.

The NFHS-5 retrospectively collected information on child death and care-related measures for 176,817 recent births by interviewing eligible mothers (15–49 years). Of them, 3,651 births that occurred within one month preceding the survey were excluded to account for censoring. Further, 1,087 deaths that occurred a few minutes or hours after deliveries were excluded as they may not be eligible for the PNC-related questions. Hence, our analytical sample consists of 172,079 recent births to eligible women in the five years preceding the NFHS-5 survey. Of them, 1,681 newborns died within one month of life.

The IIPS and ICF [7] provide detailed information on survey methods and data collection procedures.

### Outcome and exposure variable

The outcome of interest for the study is the death of a newborn within the first month of life. The exposure variables of interest for the study are measures of newborn PNC. The measures of newborn PNC included in our study are - whether the newborn received PNC within 2 days after birth, whether the newborn body temperature and umbilical cord were examined (cord-care) during the first 2 days after birth, whether the newborn breastfeeding initiated in the first one hour, and place where the newborn was delivered. The specific questions asked in NFHS-5 can be found in Supplementary: Table S1.

### Control variables

The control variables includes the time when the mother received PNC (no, within two days, in two or more days), skilled birth attendance (yes, no), mother receiving two or more tetanus injections during pregnancy (yes, no), receiving at least 100 days of iron folic acid tablets (yes, no), complications during pregnancy (yes, no) and complications during delivery (yes, no), type of delivery (c-section or normal), maternal level of schooling (no schooling, primary, secondary, higher), place of residence (rural, urban), household wealth quintile (poorest, poorer, middle, richer, richest), sex of the newborn (male, female), and type of birth (singleton birth, multiple births).

Skilled birth attendance is defined in our study as those whose delivery were assisted by doctors, nurses, auxiliary nurse midwives, and lady health visitors during delivery.

### Statistical analysis

The analysis in our study is governed by the framework of analysing the case-control study [25, 26]. Births that resulted in death within one month of life were treated as *cases*, and births that survived one month were treated as *controls*. Newborn PNC was examined among the *cases* and *controls* for different numbers of maternal ANC visits during the pregnancy. Likewise, in Carpenter et al. [27], and Singh et al. [11], if the prevalence of PNC was considerably less in *cases* than in *control*, we conclude that PNC was associated with neonatal mortality.

We used the conditional logistic regression model, a commonly used regression model to fit matched case-control data [11, 28–31]. The conditional logistic regression fits a likelihood model with a dichotomous outcome variable and differs from ordinary multiple logistic regression as it is fitted in grouped data. We used $\:{k}_{1i}:{k}_{2i}$ matching to accommodate all the births included in the survey (where $\:i$ is the $\:{i}^{th}$ matched group, $\:i=1,\:2,\:3,\dots\:,\:n$, $\:n$ is the total number of matching groups, and $\:{k}_{1}$ and $\:{k}_{2}$ represent the number of *cases* and *controls*, respectively). We used the “clogit” in STATA 16, a very flexible model for capturing the change in matching from one group to another to fit the conditional logistic model [32]. Moreover, the “clogit” model also has the flexibility to keep or eliminate the collinear variables from the regression model (See Manual [32]). Hence the model estimations are checked and free of the effects of multicollinearity. The outcome variable was coded 0 and 1 (0 indicates *control* and 1 indicates a *case*). We grouped the data for matching based on four variables: the mother’s age at birth of the newborn, previous birth intervals, birth order of the newborn, and birthsize of the newborn. Matching is necessary in a case-control study to ensure that matching factors are equally distributed between *cases* and *controls* and the confounding effects of the matching factors is mitigated [11, 25, 26, 33, 34].

The study results are appropriately weighted and adjusted for the complex survey design.

## Results

Table 1 presents the distribution of newborn PNC utilisation in the five years preceding the survey, stratified by the number of ANC visits. Around 78% of newborns received PNC services within 2 days after delivery. While, 76% of newborns’ body temperature was measured during the first 2 days after birth, 73% of newborns were examined for umbilical cord infections within the first 2 days after birth. Only about 42% of newborns were breastfed within 1 h after birth. While 62% of newborn were delivered in public healthcare facility, 28% delivered in private healthcare facility.

**Table 1:** Distribution of newborn postnatal care service utilisation in five years preceding the survey stratified by the number of maternal antenatal care visits, 2019-21

	No ANC Visits			1 to 3 ANC Visits			4 & above ANC Visits			All Sample	
	*n*	%		*n*	%		*n*	%		*n*	%
**Postnatal care**											
No	6682	54.12		16,944	26.66		15,387	13.07		39,013	20.20
Within 2 days	4302	44.82		41,730	71.64		83,938	85.44		129,970	78.26
In 2 + Days	122	1.06		1126	1.70		1848	1.49		3096	1.54
**Body temperature measured during the first two days**											
No	6134	51.77		18,009	29.72		18,831	17.85		42,974	23.96
Yes	4972	48.23		41,791	70.28		82,342	82.15		129,105	76.04
**Cord-care during the first two days**											
No	6320	53.03		19,253	31.72		22,226	21.61		47,799	26.97
Yes	4786	46.97		40,547	68.28		78,947	78.39		124,280	73.03
**Initiation of breastfeeding**											
Within 1 h	4158	32.63		22,236	35.18		47,602	46.78		73,996	41.97
In 1 + hours	6948	67.37		37,564	64.82		53,571	53.22		98,083	58.03
**Place where newborn delivered**											
Home / Elsewhere	3306	29.77		8261	13.82		5702	5.64		17,022	9.89
Public facility	5893	53.06		38,065	63.65		62,493	61.77		106,479	61.88
Private facility	1907	17.17		13,473	22.53		32,978	32.60		48,578	28.23

PNC utilisation increases with increasing number of maternal ANC visits. Among those births that occurred to women who had no ANC visits, only 45% newborns received PNC within 2 days after birth. Whereas 72% and 85% of newborns to mothers with 1 to 3 ANC visits and four or more ANC visits, respectively, received PNC. Likewise, body temperature was measured for 82% of newborns to mothers who had four or more ANC visits. In comparison, body temperature was measured for only 48% of newborns to mothers who did not attend any ANC visits. While 78% of newborns to mothers who had four or more ANC visits received cord care, only 47% of newborns to mothers without ANC visits received cord care. Newborns breastfeed within 1 h after birth ranged from 33% among those births that occurred to women who had no ANC visits to 47% among those births that occurred to women who had four or more ANC visits. The percentage of newborn delivered in public health facility ranged from 53% among births occurred to women who had no ANC visits to 64% among births occurred to women who had 1 to 3 ANC visits. Similarly, among births occurred to women who had no ANC visits, 17% were delivered in private facility, and among births that occurred to women who had four of more ANC visits, 33% delivered in private facility.

Table 2 presents the prevalence of newborn PNC utilisation by the survival status stratified by the number of ANC visits. Only 64.8% (95% CI = [61.9, 67.8]) of newborns who died during the first month of life received PNC within two days after delivery, compared to 78.4% (95% CI = [78.0, 78.8]) among newborns who survived one month of life. While body temperature was measured during the first two days of life for 52.0% (95% CI = [48.8, 55.2]) of the newborns who died during the neonatal period, it was measured for 76.3% (95% CI = [75.8, 76.7]) of newborns who survived the neonatal period. Similarly, a higher percentage of babies were examined for infections in the umbilical cord during the first two days of life among those who survived the neonatal period (73.3%; 95% CI = [72.8, 73.8]) compared with those who died during the neonatal period (48.6%; 95% CI = [45.4, 51.8]). The percentage of newborns breastfed within one hour after birth among those who survived the neonatal period is twice that of those who died during the neonatal period (42.2%; 95% CI = [41.6, 42.7] Vs 21.8%; 95% CI = [19.0, 24.5]).

**Table 2:** Newborn postnatal care utilisation in five years preceding the survey by the survival status during the neonatal period stratified by the number of maternal antenatal care visits, 2019-21

	No ANC visits				1 to 3 ANC visits				4 & above ANC visits				All Sample		
	Survivors		Deaths		Survivors		Deaths		Survivors		Deaths		Survivors		Deaths
	% (95% CI)		% (95% CI)		% (95% CI)		% (95% CI)		% (95% CI)		% (95% CI)		% (95% CI)		% (95% CI)
**Postnatal care**															
Within 2 days	45.0 (43.2, 46.7)		36.8 (27.5, 46.0)		71.8 (71.1, 72.4)		61.2 (56.6, 65.7)		85.5 (85.1, 85.9)		74.5 (70.4, 78.5)		78.4 (78.0, 78.8)		64.8 (61.9, 67.8)
**Body temperature measured during the first two days**															
Yes	48.6 (46.8, 50.3)		29.7 (21.4, 37.9)		70.6 (69.9, 71.3)		47.3 (42.6, 52.0)		82.3 (81.8, 82.8)		61.2 (56.6, 65.9)		76.3 (75.8, 76.7)		52.0 (48.8, 55.2)
**Cord-care during the first two days**															
Yes	47.3 (45.6, 49.1)		27.6 (19.4, 35.8)		68.6 (67.8, 69.3)		46.1 (41.3, 50.8)		78.6 (78.0, 79.1)		55.6 (50.8, 60.3)		73.3 (72.8, 73.8)		48.6 (45.4, 51.8)
**Initiation of breastfeeding**															
Within 1 h	32.9 (31.2, 34.6)		16.5 (10.1, 23.0)		35.4 (34.6, 36.2)		17.7 (14.2, 21.1)		46.9 (46.3, 47.6)		26.5 (21.8, 31.1)		42.2 (41.6, 42.7)		21.8 (19.0, 24.5)
**Place where newborn delivered**															
Public facility	53.1 (51.5, 54.6)		53.5 (44.1, 63.0)		63.8 (63.1, 64.5)		53.2 (48.5, 57.9)		61.7 (61.1, 62.3)		66.7 (62.3, 71.2)		61.9 (61.4, 62.4)		59.7 (56.6, 62.9)
Private facility	17.1 (16.0, 18.3)		18.3 (11.2, 25.4)		22.4 (21.7, 23.1)		31.5 (27.0, 36.0)		32.7 (32.1, 33.3)		25.3 (21.2, 29.4)		28.2 (27.7, 28.7)		27.0 (24.2, 29.9)

Note: ANC - antenatal care; CI - confidence interval; NA - Not Applicable (no observation)

A similar pattern was observed irrespective of the number of ANC visits. However, the utilisation of newborn PNC within two days after birth among the survivors notably increases with increasing number of maternal ANC visits.

There is no notable difference in the prevalence of newborns delivered in public healthcare facilities among those who survived the neonatal period and those who died during the neonatal period. A similar pattern could be seen among those delivered in private healthcare facilities. However, the prevalence of newborns delivered in both public and private healthcare facilities increases with increasing number of maternal ANC visits.

Additionally, we estimated the prevalence of newborn PNC utilisation by the place where newborn was delivered (See Supplementary: Table S2). While 12.6% of newborns in public health facilities do not receive PNC within 2 days or 2 + days, only 8.9% of newborns delivered in private healthcare facilities do not receive the required PNC. Only 21.6% and 19.9% of newborns delivered in public and private healthcare facilities did not measure their body temperature during the first two days after delivery. Similarly, only 24.8% and 24.7% of newborns delivered in public and private healthcare facilities, respectively, did not receive cord-care. And 44.8% and 38.3% of newborns delivered in public and private healthcare facilities were not breastfed within 1 h after delivery.

The prevalence of newborns receiving PNC within 2 days after delivery, having body temperature measured and receiving cord care within the first two days after delivery increases with the number of ANC visits, irrespective of whether the newborns was delivered in a public or private healthcare facility. However, the prevalence of initiation of breastfeeding within 1 h after delivery decreases with increasing number of ANC visits. A higher prevalence of early breastfeeding initiation among newborns delivered in the public healthcare facility was observed.

Table 3 presents the conditional logistic regression estimates of the effects of newborn PNC utilisation on neonatal mortality adjusted for selected maternal and child characteristics. Compared to those infants who did not receive PNC, infants who received PNC within two days (OR = 0.69; 95% CI = [0.59, 0.81]) or in two or more days (OR = 0.39; 95% CI = [0.22, 0.69]) after birth have a lower risk of neonatal mortality. Similarly, infants whose body temperature was measured (OR = 0.60; 95% CI = [0.52, 0.69]) and whose umbilical cord was examined (OR = 0.58; 95% CI = [0.50, 0.67]) during the first two days of birth have a lower risk of neonatal mortality. However, infants who were breastfed one hour or later after delivery have a higher risk of neonatal mortality than those who were breastfed within one hour of delivery (OR = 2.32; 95% CI = [2.06, 2.62]).

**Table 3:** Conditional logistic regression estimates of the effects of newborn postnatal care utilisation on neonatal mortality

		No ANC Visits ^a^					1 to 3 ANC Visits ^b^					4 & above ANC Visits ^c^					All Sample ^d^			
		OR ^#^	*p*-value	95% CI			OR ^#^	*p*-value	95% CI			OR ^#^	*p*-value	95% CI			OR ^#^	*p*-value	95% CI	
**Postnatal care**																				
No ^®^																				
Within 2 days		0.90	0.660	0.57	1.43		0.64	< 0.001	0.51	0.81		0.78	0.038	0.61	0.99		0.69	< 0.001	0.59	0.81
In 2 + Days		*NA*					0.43	0.033	0.19	0.93		0.42	0.040	0.18	0.96		0.39	0.001	0.22	0.69
**Body temperature measured during the first two days**																				
No ^®^																				
Yes		0.77	0.252	0.50	1.20		0.61	< 0.001	0.49	0.75		0.59	< 0.001	0.47	0.72		0.60	< 0.001	0.52	0.69
**Cord-care during the first two days**																				
No ^®^																				
Yes		0.61	0.026	0.39	0.94		0.57	< 0.001	0.46	0.71		0.58	< 0.001	0.47	0.71		0.58	< 0.001	0.50	0.67
**Initiation of breastfeeding**																				
Within 1 h ^®^																				
In 1 + hours		2.50	< 0.001	1.68	3.72		2.40	< 0.001	1.98	2.93		2.38	< 0.001	2.02	2.79		2.32	< 0.001	2.06	2.62
**Place where newborn delivered**																				
Home / Elsewhere ^®^																				
Public facility		0.75	0.317	0.42	1.32		0.59	0.004	0.42	0.84		0.50	< 0.001	0.34	0.73		0.54	< 0.001	0.42	0.68
Private facility		0.52	0.071	0.26	1.06		0.40	< 0.001	0.27	0.59		0.51	0.002	0.33	0.77		0.45	< 0.001	0.34	0.58

Note:

ANC - antenatal care; CI - Confidence Interval; ^®^ - Reference category; *NA* - Not Applicable due to no cases.

^**a**^ 435 groups (4,250 observations) dropped because of all positive or all negative outcomes.

^**b**^ 395 groups (6,809 observations) dropped because of all positive or all negative outcomes.

^**c**^ 388 groups (6,604 observations) dropped because of all positive or all negative outcomes.

^**d**^ 2587 groups (34,292 observations) dropped because of all positive or all negative outcomes.

^**#**^***control variables***: time when mother received the postnatal check, skilled birth attendance, maternal level of schooling, household wealth quintile, place of residence, sex of the newborn, type of birth, mother receiving 2 + tetanus injections during pregnancy, mother’s receiving at least 100 days of iron folic acid tablets, complications during pregnancy and delivery, and type of delivery.

***Matching variables***: maternal age at birth, previous birth interval, birth order, birthweight.

Infants who received PNC within two days of birth and were born to mothers with 1 to 3 ANC visits (OR = 0.64; 95% CI = [0.51, 0.81]) and four or more ANC visits (OR = 0.78; 95% CI = [0.61, 0.99]) have a lower risk of dying within one month of life. The risk of neonatal mortality was also lower among babies whose body temperature was measured and born to women who had 1 to 3 ANC visits (OR = 0.61; 95% CI = [0.49, 0.75]) and four or more ANC visits (OR = 0.59; 95% CI = [0.47, 0.71]). Similarly, the risk of newborn death within one month of birth was lower among babies whose umbilical cord was examined, irrespective of the number of ANC visits. Similarly, babies delivered in either public or private healthcare facilities were observed to have a lower risk of dying within one month of life, irrespective of the number of ANC visits. And, babies who were not breastfed within one hour after delivery were observed to have a higher risk of dying within one month of life, irrespective of the number of ANC visits.

## Discussion

Our study explored the impact of newborn PNC utilisation on neonatal mortality with varying numbers of mothers’ ANC visits, employing a matched case-control study framework: births that ended in death within one month of birth and survived one month after birth were considered as *cases* and *controls* respectively. Matching was essential to ensure that matching factors were equally distributed between *cases* and *controls* [34], adjusting the associations between the matching factors and neonatal mortality, thereby mitigating the confounding effects of matching factors’ [11, 25, 26, 33, 34]. Considering India’s lower rate of maternal four or more ANC and PNC attendance and its cohesive relationship with newborn health and wellbeing, understanding this relationship will aid in the effort to reduce neonatal mortality, thereby helping achieve SDG goal 3.2 of reducing neonatal mortality to 12 deaths per 1,000 live births.

The risk of newborn deaths during the neonatal period was observed to be closely related to newborn PNC utilisation. We observed a relatively higher PNC among newborns who survived the first month of life than those who died. Newborns receiving PNC within two days or more than two days of birth are less likely to die during the neonatal period. Similarly, births that occurred to mothers who had attended more ANC visits and received PNC within two days after birth have a lower risk of dying during the neonatal period. In addition, the percentage of newborns receiving PNC within two days after birth notably increases with the increase in mothers’ ANC visits. These findings have decisive policy implications, particularly as the distribution of maternal healthcare utilisation in India is not uniform across the reproductive period [7]. In fact, Fekadu et al. [16] noted a higher number of ANC attendance has a multiplicative effect on practising PNC for both mother and newborn. Literature based on LMICs reported a similar relationship and called on the need for the policymakers, programme planners and stakeholders to develop and implement strategies to increase the number of ANC visits to strengthen the use of PNC, which will aid in neonatal mortality reduction [16, 35–37].

Acknowledging the need for and importance of maternal and child healthcare services during pregnancy and delivery, stakeholders worldwide have increased their focus on maternal and child healthcare. However, Marchant et al. [38] noted the importance of shifting the focus from global to local initiatives that strengthen the healthcare systems in the regions where they are most required. And, India is a particular case where it has prioritised maternal and child healthcare and launched a national strategy named RMNCH + A to accelerate access to maternal healthcare and child survival in early 2013 [10]. RMNCH + A was a strategy to guide the stakeholders, programme planners and implementers to understand better the comprehensive approach to improving India’s maternal and child healthcare system under the National Rural Health Mission (NRHM), now integrated into the National Health Mission (NHM). Since the launch of NRHM in 2005, there has been substantial development in the maternal and child healthcare infrastructure and the expansion of programme management capacity. Although these strategies helped increase the accessibility, availability and affordability of maternal healthcare services, the neonatal mortality rate, which contributes to about 60% of under-five deaths in India, remains high, with 25 deaths per 1,000 live births in 2019-21 [7]. The disproportionate distribution of maternal and child healthcare utilisation during the reproductive period [7], coupled with the high neonatal mortality, calls for a new or at least an update in the approach (covering all three levels of care, namely, antenatal, delivery, and postnatal) to implement the available policies and programs more effectively and pace up the race to increase maternal and child healthcare service utilisation. Enhancing the availability and accessibility of ANC services and safe delivery, together with focused financial aid and health promotion programmes, identifying the pockets with poor access to healthcare (for instance, rural areas) would help increase PNC utilisation [19, 39]. For instance, stakeholders in India can focus more on improving its lower rate of maternal four or more ANC visits (59% [7]) and PNC visits (61% [7]), along with other measures, thereby aiding in both maternal and child healthcare. On the other hand, IIPS & ICF [7] noted a notable gap in the newborn PNC utilization among babies born in a healthcare facilities (81%) and babies born at home or elsewhere (31%). In fact, our study highlighted a higher prevalence of newborns PNC utilisation among births that occurred in either public or private healthcare facilities and these newborns also have a lower risk of neonatal mortality.

Promoting continuous monitoring of newborn body temperature and examining the umbilical cord in the early days of life could also help reduce neonatal mortality. Bacterial infections contributes to about 0.7 million neonatal deaths, yearly [40, 41]. Many of these infections are due to improper cord-care practices, as they vary from one community to another due to their different cultural and traditional practices [42]. Improper and unhygienic cord-care practices could lead to bacterial colonisation in the umbilicus, allowing the bacteria to enter the newborn’s bloodstream, leading to different illnesses and even death. Therefore, practising hygienic cord-care is a must to prevent deadly neonatal tetanus for a newborn who has not developed passive immunity [42–44]. Our study also found a similar relationship between cord-care and neonatal mortality. We found a lower risk of neonatal mortality among those newborns whose umbilical cord was examined within two days after birth by the health care provider regardless of the number of ANC visits. Similarly, the risk of newborn deaths was lower among babies whose body temperature was measured within two days after birth. Boundy et al. [45], Cunningham et al. [46], Lawn et al. [47], Singh et al. [11], and WHO Immediate KMC Study Group [48] also noted a similar finding where they reported that births occurred to women who had received advice about *kangaroo care* (a type of infant care that involves skin-to-skin contact with the mother or other caregiver) or *keeping the baby warm* after birth during the antenatal period and baby who received immediate *kangaroo care* had a lower risk of dying in the first month of life. In addition, we also found that the percentage of newborns receiving cord care and body temperature measured during the first two days of birth increases with the increase in the number of maternal ANC visits.

Initiation of breastfeeding within an hour after birth was observed to be protective against neonatal mortality, irrespective of maternal ANC visits. The tendency to breastfeed their newborns within one hour after delivery was considerably higher among those births that occurred to mothers who had a higher number of maternal ANC visits. Our finding aligns with the existing literature [2, 49, 50]. Indeed, Edmond et al. [50], Debes et al. [49], and Khan et al. [51] recommended early initiation of breastfeeding as a simple and cost-effective measure that can potentially reduce the risk of neonatal deaths.

While we discuss the important contribution of the findings of our study extensively, it is also important to note the strengths and limitations of our study. The primary strength of the study is the use of India’s most recent population-representative survey (NFHS-5), which primarily focuses on maternal and child health-related indicators [7]. The large sample size (0.8 million recent births) allows us to study and discuss the association between newborn PNC utilisation and neonatal mortality at different levels of maternal ANC visits. NFHS-5, for the first time, collected information on cord-care in a large-scale survey [7]. Using this information, we could show the relationship between cord-care practices and neonatal mortality for the first time using a nationally representative sample. Finally, the matched case-control study framework adopted in the study makes the estimates of the relationship between newborn PNC and neonatal mortality more robust by mitigating the confounding effects of the matching factors.

Despite these strengths, we acknowledge a few limitations, too. NFHS-5 retrospectively collected information on recent births’ care-related characteristics by interviewing eligible mothers (15–49 years). Due to its retrospective nature, recall bias may be associated with this information. Past studies have shown the effectiveness of *kangaroo care* in reducing early newborn deaths [11, 45, 47]. However, we could not precisely capture the effect of *kangaroo care* on neonatal mortality, as NFHS-5 does not ask a direct question about keeping the baby warm. We use the information on whether the baby’s body temperature was measured during the first two days after birth, though the survey does not specify how frequently the baby’s body temperature was measured, as a proxy measure for *kangaroo care*, which was found to be a potential factor that can help in neonatal mortality reduction.

## Conclusion

Considering the cohesive nature of the relationship between neonatal mortality and maternal and child healthcare utilisation, strategic planning and management of the existing policies and programmes related to accessibility, availability, and affordability of maternal and child healthcare services is needed to achieve goal 3.2 of the SDGs by 2030 successfully. Promoting cost-effective measures such as continuous monitoring of the baby’s body temperature and umbilical cord care could also effectively help reduce neonatal mortality.

## Electronic supplementary material

Below is the link to the electronic supplementary material.

Supplementary Material 1

## Data Availability

The NFHS-5 data is available for public use in DHS data repository https://dhsprogram.com/data/dataset/India_Standard-DHS_2020.cfm?flag=1. It can be accessed from the data repository by registering and submitting a data request application. The user will be provided access to the datasets once the application is approved.

## Abbreviations

ANC: Antenatal Care
CI: Confidence Interval
JSSK: Janani Sishu Suraksha Karyakram
LMICs: Low-and-middle-income countries
NFHS-5: Fifth National Family Health Survey
NHM: National Health Mission
NRHM: National Rural Health Mission
OR: Odds Ratio
PNC: Postnatal Care
SDGs: Sustainable Development Goals
