# Supplementary material for: The nexus between maternal antenatal care attendance, newborn postnatal care and neonatal mortality in India: a matched case-control study
Source: BMC Pregnancy Childbirth. 2024 Oct 22;24:691. doi: 10.1186/s12884-024-06881-6 (PMC11520158; doi:10.1186/s12884-024-06881-6)
Supplement: Supplementary file 1 — Supplementary Material 1 [file 12884_2024_6881_MOESM1_ESM.docx]

**Supplementary**

**Table S1**: Questions in NFHS-5 (2019-21) for care-related exposure variables considered in the study.

| **Question No.^#^** | **Question** | **Response** | | | | |
| --- | --- | --- | --- | --- | --- | --- |
|  |  |  | | | | |
| 490 | How many hours, days or weeks after the birth of newborn did the first check take place. | Hours after birth | |  |  |  |
|  |  | Days after birth | |  |  |  |
|  |  | Weeks after birth | |  |  |  |
|  |  |  | | | | |
| 494 | During the first two days after newborn birth, did any healthcare provider do the following |  | | | | |
|  | 1. Examine the cord? |  | Yes / No | | |  |
|  | 1. Measure body temperature? |  | Yes / No | | |  |
|  |  |  | | | | |
| 499c | How long after birth did you start breastfeeding | Immediately | | 000 | |  |
|  |  | Hours | |  |  |  |
|  |  | Days | |  |  |  |
|  |  |  | |  |  |  |

**Note**: ^#^ “Question No.” are the actual question number provided in the NFHS-5 women’s questionnaire <http://rchiips.org/NFHS/NFHS5/schedules/NFHS-5Womans.pdf>

**Table S2**: Newborn postnatal care utilisation in five years preceding the survey by place of delivery, 2019-21

|  | **No ANC visits** | | |  | **1 to 3 ANC visits** | | |  | **4 & above ANC visits** | | |  | **All Sample** | | |
| --- | --- | --- | --- | --- | --- | --- | --- | --- | --- | --- | --- | --- | --- | --- | --- |
|  | **Public facility** |  | **Private facility** |  | **Public facility** |  | **Private facility** |  | **Public facility** |  | **Private facility** |  | **Public facility** |  | **Private facility** |
|  | **% (95% CI)** |  | **% (95% CI)** |  | **% (95% CI)** |  | **% (95% CI)** |  | **% (95% CI)** |  | **% (95% CI)** |  | **% (95% CI)** |  | **% (95% CI)** |
| **Postnatal care** |  |  |  |  |  |  |  |  |  |  |  |  |  |  |  |
| No | 36.5 (34.4, 38.7) |  | 29.0 (25.8, 32.2) |  | 15.8 (15.2, 16.4) |  | 12.3 (11.4, 13.2) |  | 8.6 (8.2, 9) |  | 6.5 (6, 6.9) |  | 12.6 (12.2, 13.0) |  | 8.9 (8.4, 9.3) |
| Within 2 days | 62.0 (59.9, 64.2) |  | 69.4 (66.1, 72.6) |  | 82.3 (81.6, 82.9) |  | 85.5 (84.5, 86.5) |  | 89.7 (89.3, 90.1) |  | 92.1 (91.7, 92.6) |  | 85.7 (85.3, 86.0) |  | 89.5 (89.0, 90.0) |
| In 2+ Days | 1.5 (1.1, 1.9) |  | 1.6 (0.9, 2.4) |  | 1.9 (1.7, 2.1) |  | 2.2 (1.8, 2.6) |  | 1.7 (1.5, 1.8) |  | 1.4 (1.2, 1.6) |  | 1.7 (1.6, 1.9) |  | 1.6 (1.5, 1.8) |
| **Body temperature measured during the first two days** | | | |  |  |  |  |  |  |  |  |  |  |  |  |
| No | 43.8 (41.5, 46) |  | 40.8 (37, 44.6) |  | 26.4 (25.7, 27.2) |  | 26.1 (24.7, 27.5) |  | 16.8 (16.2, 17.3) |  | 16.3 (15.6, 17.1) |  | 21.6 (21.1, 22.1) |  | 19.9 (19.2, 20.6) |
| Yes | 56.2 (54, 58.5) |  | 59.2 (55.4, 63) |  | 73.6 (72.8, 74.3) |  | 73.9 (72.5, 75.3) |  | 83.2 (82.7, 83.8) |  | 83.7 (82.9, 84.4) |  | 78.4 (77.9, 78.9) |  | 80.1 (79.4, 80.8) |
| **Cord-care during the first two days** | |  |  |  |  |  |  |  |  |  |  |  |  |  |  |
| No | 46.5 (44.3, 48.8) |  | 45.7 (41.8, 49.7) |  | 28.6 (27.8, 29.4) |  | 31.3 (29.3, 33.3) |  | 20.6 (20.0, 21.2) |  | 21.0 (20.2, 21.8) |  | 24.8 (24.2, 25.3) |  | 24.7 (23.9, 25.5) |
| Yes | 53.5 (51.2, 55.7) |  | 54.3 (50.3, 58.2) |  | 71.4 (70.6, 72.2) |  | 68.7 (66.7, 70.7) |  | 79.4 (78.8, 80.0) |  | 79.0 (78.2, 79.8) |  | 75.2 (74.7, 75.8) |  | 75.3 (74.5, 76.1) |
| **Initiation of breastfeeding** |  |  |  |  |  |  |  |  |  |  |  |  |  |  |  |
| Within 1 hour | 35.8 (33.6, 38.0) |  | 28.4 (25.1, 31.7) |  | 37.7 (36.9, 38.6) |  | 31.1 (29.0, 33.1) |  | 49.8 (49.0, 50.5) |  | 41.7 (40.7, 42.7) |  | 44.8 (44.2, 45.4) |  | 38.3 (37.4, 39.3) |
| In 1+ hours | 64.2 (62.0, 66.4) |  | 71.6 (68.3, 74.9) |  | 62.3 (61.4, 63.1) |  | 68.9 (66.9, 71.0) |  | 50.2 (49.5, 51.0) |  | 58.3 (57.3, 59.3) |  | 55.2 (54.6, 55.8) |  | 61.7 (60.7, 62.6) |

***Note***: ANC - antenatal care; CI - confidence interval
